# Formalin and 2.5% Glutaraldehyde/2% Paraformaldehyde in 0.1 M Cacodylate Buffer Inactivation Protocols to Ensure the Proper Fixation of Positive Sense RNA Viruses and Genomic Material Prior to Removal from Containment

**DOI:** 10.3390/mps7060105

**Published:** 2024-12-21

**Authors:** Lauren E. Panny, Ashley E. Piper, Christina L. Gardner, Crystal W. Burke

**Affiliations:** Virology Division, United States Army Medical Research Institute of Infectious Diseases (USAMRIID), Fort Detrick, Frederick, MD 21702, USA; lauren.e.panny.ctr@health.mil (L.E.P.); ashley.e.piper2.civ@health.mil (A.E.P.); christina.l.gardner8.ctr@health.mil (C.L.G.)

**Keywords:** positive-strand RNA virus, inactivation, formalin, glut/PFA, EEEV, eastern equine encephalitis virus, validation

## Abstract

Recommendations released by the CDC in 2023 address the need to demonstrate that the RNA genome of positive-strand RNA viruses is inactivated in addition to viral particles. This recommendation is in response to the similarities between host mRNA and the viral genome that allow the viral RNA to be used as a template by host replication mechanisms to produce infectious viruses; therefore, there is concern that through artificial introduction into host cells, active positive-strand RNA genomes can be utilized to produce infectious viruses out of a containment facility. Utilizing 10% formalin for 7 days or 2.5% glutaraldehyde/2% paraformaldehyde in 0.1 M cacodylate buffer (glut/PFA) for 2 days to fix eastern equine encephalitis virus (EEEV)-infected non-human primate (NHP) brain tissue was found to effectively inactivate EEEV particles and genomic RNA. The methods assessed in this paper outline an effective means to validate both genomic RNA and viral particle inactivation.

## 1. Introduction

Viral agents and associated components (ex., genomic material, viral proteins, etc.) studied in a biocontainment suite commonly require transfer to a laboratory of lower containment level for processing purposes. Removal of these infectious materials requires inactivation techniques that are extensively validated and approved by the Federal Select Agent Program (FSAP) to minimize the potential of release [1]. These inactivation techniques should thoroughly address all potential scenarios that would allow for virus propagation to occur outside of the biocontainment facility; therefore, the genome of positive-strand RNA viruses must be proven inactivated as well since the genome itself can be infectious through intentional introduction into the cell using methods such as electroporation and transfection [1].

**Figure 1:**
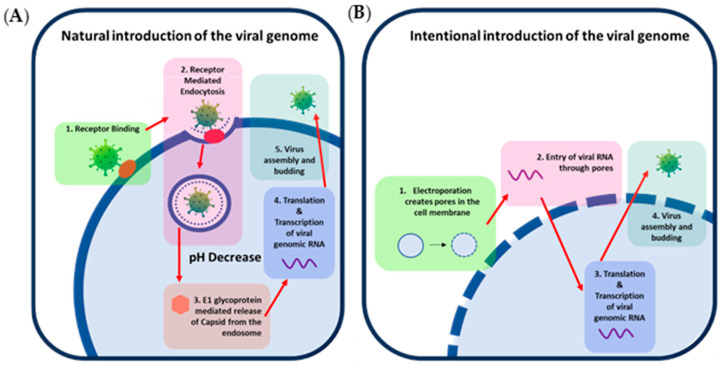
Mechanisms of introduction of positive-sense viral RNA into cells. (**A**) Depicts infection of a host cell with a natural EEEV particle. The virus entry protein, E2, binds to a host receptor (1) that allows for entry into the host cell via endocytosis (2). Within the endosome, the pH decreases, allowing for conformational changes of the entry and fusion proteins, enabling the fusion protein, E1, to create a pore within the endosome, resulting in the release of the viral capsid into the cytosol (3). The capsid is then disassembled by the host ribosome, and viral genomic RNA is released, transcribed, and translated into viral proteins (4). Structural proteins and genomic RNA then assemble and bud at the peripheral membrane to form progeny virions. (**B**) Depicts intentional introduction of the viral genomic RNA via electroporation for comparison. (1) Electroporation creates pores in the peripheral membrane, (2) allowing artificially introduced EEEV genomic RNA to enter the cell without the viral structural proteins typically needed for entry and fusion steps. (3) Replication, transcription, and translation then occur in the same manner as natural virus infection, (4) producing progeny virions that can infect and replicate in the same manner as 1A.

The infectivity of the genome of positive-strand RNA viruses can be best demonstrated through an understanding of positive-strand RNA virus replication. The alphavirus, eastern equine encephalitis virus (EEEV), can be used as a model positive-strand RNA virus to demonstrate replication. EEEV replication classically begins with receptor-mediated entry into the cell that initiates endocytosis [2]. As pH drops within the endosome, fusion is initiated by the E1 glycoprotein, allowing for the release of the capsid and viral genome. Breakdown of the capsid by host ribosomes then releases the genome into the cytoplasm [3,4]. Non-structural polyproteins nsP1234 and nsP123 are translated by the host ribosomes and other host translational proteins from the positive-strand RNA genome released from the capsid [5]. NsP1234 self-cleaves into the nsP123 polyprotein and nsP4 viral non-structural protein forming a complex that allows for the production of negative-strand RNA. Further cleavage events produce the non-structural proteins NsP1, NsP2, NsP3, and NsP4 that form a complex that transcribes genomic positive-strand RNA from negative-strand genomic RNA [5,6]. Negative-strand RNA is also used as a template to produce sub-genomic RNA that is needed to produce the structural proteins necessary for building the progeny viral particles [5,6,7]. These structural proteins are processed and are then transported to the host cell’s peripheral membrane for packaging of the capsid-encapsulated genomic RNA creating a new infectious virus [5,6]. This demonstrates that through bypassing of entry and fusion steps that require viral proteins, transcription of the viral RNA can occur through utilization of the host transcription mechanisms (Figure 1); therefore, artificial introduction of infectious genomic RNA into the cell would effectively produce infectious virus.

In recent years, the CDC and FDA select agent program released guidance that “select agent and toxin nucleic acids that are inherently infectious and are immediate precursors to virus production (i.e., the nucleic acids are capable of generating infectious forms of a regulated virus by utilizing host polymerases but without the need for any additional exogenous factors [proteins, nucleic acids, etc.])” are FSAP regulated; therefore, all positive-strand RNA viruses and their associated nucleic acids classified as BSAT (Table 1) must utilize inactivation protocols that prove genomic RNA is also inactivated [8]. The research associated with this paper covers two techniques for the inactivation of positive-strand viruses, 10% formalin fixation and fixation with 2.5% glutaraldehyde/2% paraformaldehyde in 0.1 M cacodylate buffer (glut/PFA), using eastern equine encephalitis virus (EEEV) as a model agent. Formalin was explored since it is a universally used fixative within the pathology field for techniques such as histochemistry and immunohistochemistry [9]. Glutaraldehyde and paraformaldehyde in cacodylate buffer, on the other hand, is a commonly utilized fixative for electron microscopy techniques [10,11,12]. Both of these fixatives inactivate samples by creating covalent bonds between tissue proteins, allowing for the structure of the tissue and cells to remain intact while still successfully inactivating the sample enabling microscopic imaging similar to unfixed samples [13]. Additionally, inactivation with these reagents provides a rapid, straightforward, and affordable means of fixing samples [9].

## 2. Materials and Methods (Figure 2)

### 2.1. Non-Human Primate Infection

Cynomolgus macaques of Indochinese origin were administered 4.5 × 10^8^ PFU of EEEV V105-00210 using a mucosal atomization device to evaluate disease after large particle exposure. On day 6 post-infection, two NHPs were moribund and met euthanasia criteria. Brain tissues were collected during necropsy and stored at −80 °C for future research purposes and to maximize utilization of the animals. Segments of this tissue were used for the inactivation studies described here.

**Figure 2:**
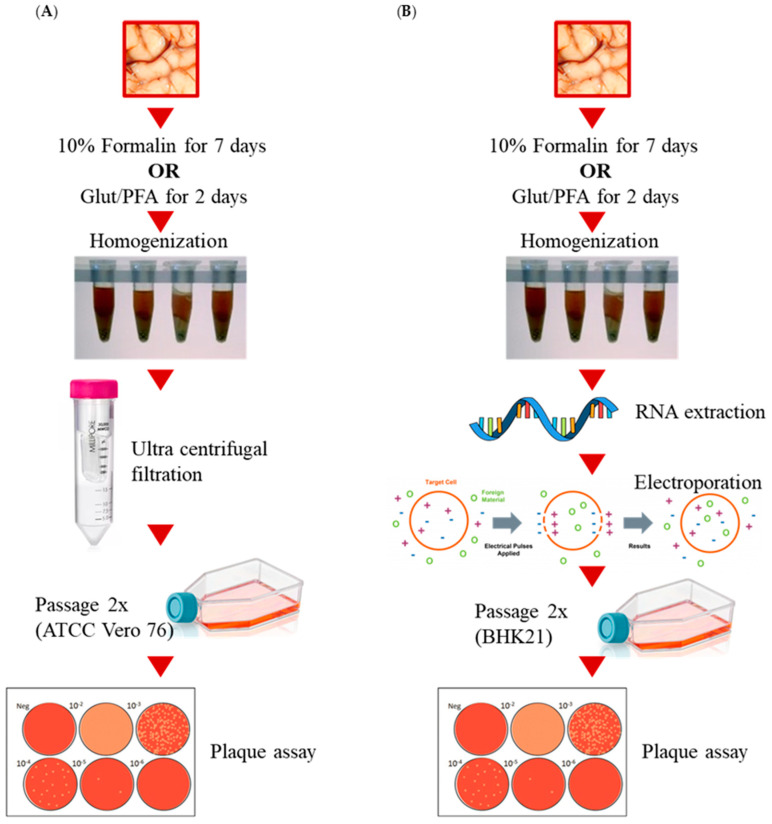
Schematics depicting the workflow process for testing (**A**) viral particle inactivation and (**B**) viral RNA inactivation.

### 2.2. Inactivation of Brain Tissue Samples

Brain samples were thawed at room temperature and then cut into approximately 1 × 1 × 1 cm^3^ cubes for formalin samples and 0.5 × 0.5 × 0.5 cm^3^ cubes for glut/PFA samples. Three experiments were performed with three replicates of formalin or glut/PFA inactivated cubes per experiment. Cubes for inactivation were submerged in 10% formalin at room temperature for 7 days or glut/PFA for 2 days. One cube per experiment was also placed in the −80 °C ± 10 °C freezer without fixative to be used as a positive control.

### 2.3. Tissue Processing of Formalin Inactivated Samples and Glut/PFA Inactivated Samples

Samples were removed from fixative (either formalin or glut/PFA) or removed from the freezer and thawed. Each cube was then washed three times with PBS (Gibco, Miami, FL, USA, Cat: 10010-023). After three washes, the cube was minced using a scalpel. The minced tissue was then washed with PBS seven more times. Samples were then placed in tissue dissociator gentleMACS M tubes (Miltenyi Biotech, Gaithersburg, MD, USA, Cat: 130096335) with 10 mL of inactivation testing media (MEM + 5% FBS + 2% pen/strep + 1% HEPES). Tissues were then homogenized using a GentleMacs homogenizer (Miltenyi Biotech, Gaithersburg, MD, USA, Cat:130-093-235) using the programmed RNA_02 setting. Samples were then placed in a new tube and centrifuged at 2000× *g* for ten minutes. Supernatants were then transferred to a new tube and centrifuged again. Supernatants were then removed and strained through a 40 µm cell strainer (BD Falcon, Franklin Lakes, NJ, USA, Cat:352340) into a new tube to remove tissue debris. Samples were then frozen at −80 °C ± 10 °C.

The following day, samples were thawed and transferred to a 100 K Ultra-15 centricon filtration system (Millipore, Jaffrey, New Hampshire, USA, Cat: UFC910024) to remove formalin or glut/PFA without removing virus. Positive column controls were prepared from stock virus diluted to 10 PFU/mL and 1 PFU/mL. A total of 10 mL of each positive column control was then added to centricon filtration systems. All tubes were spun at 2500× *g* for 20 min at room temperature. Flow through was discarded, and supernatants were transferred to a new 100 K Ultra-15 centricon filtration system. PBS was added to each sample to bring the volume to 10 mL. This step was repeated a total of four times with supernatants being transferred to a conical after the final centrifugation step (no PBS added). Volumes were then assessed, and smaller volumes were brought up to equal the greatest volume collected with inactivation testing media. Example: 3 mL, 5 mL, and 0.5 mL of supernatant were collected; therefore, all tubes would be brought up to 5 mL through addition of inactivation testing media. Samples were then frozen at −80 °C ± 10 °C.

### 2.4. Safety Testing Passage for Formalin and Glut/PFA Samples

Frozen supernatants from Section 3 were thawed and brought up to 22 mL with inactivation testing media. The 22 mL samples were then transferred to T75 flasks of ATCC Vero 76 cells (ATCC, Manassas, VA, USA, Cat: CRL-1587). In addition, safety test controls were made by diluting stock virus to 100 PFU/mL, 10 PFU/mL, and 1 PFU/mL. A total of 1 mL of diluted virus of each PFU was then added to 21 mL of inactivation media. The total 22 mL for each control was then transferred to T-75 flasks of ATCC Vero 76 cells. A final T-75 flask of ATCC Vero 76 had 22 mL of fresh inactivation media added as overlay to use as a negative safety control. Flasks were then incubated at 37 °C ± 2 °C and 5 ± 1% CO_2_ for 72 h. Cell CPE was checked daily, and control supernatants of cells with CPE greater than 80% were collected prior to the 72 hpi timepoint. At greater than 72 h post-infection, supernatants of inactivated groups and any remaining controls were collected. Supernatants were then centrifuged at 500× *g* for five minutes to remove cell debris. For all samples at time of collection, supernatants were transferred to fresh tubes and were frozen at −80 °C ± 10 °C freezer or immediately used for passage 2 experiments.

For passage 2, any frozen supernatants were thawed at ambient temperature. A total of 11 mL of each sample was then added to 11 mL of inactivation testing media. All 22 mL was then transferred to a T-75 flask of ATCC Vero 76 cells and allowed to incubate at 37 °C ± 2 °C and 5 ± 1% CO_2_ for 72 h. Cell CPE was checked daily, and control supernatants of cells with CPE greater than 80% were collected prior to the 72 hpi timepoint. At greater than 72 h post-infection or within a few hours later (never earlier), supernatants of inactivated groups and any remaining controls were collected. For all samples at time of collection, supernatants were then centrifuged at 500× *g* for five minutes to remove cell debris. Supernatants were transferred to fresh tubes and were frozen at −80 °C ± 10 °C.

### 2.5. Plaque Assay for Infectious Virus in Formalin and Glut/PFA Inactivated Samples

Pass 2 samples were thawed at ambient temperature. Samples that were determined to have 0 CPE during safety testing were plaqued neat (undiluted). Samples with observed CPE during safety testing (passage 1 or 2) were diluted 1:10 in virus diluent media from 10^1^ to 10^9^. Stock virus was also diluted in this fashion as a positive control. Virus diluent media was used on cells as a negative control for the assay. Six well plates seeded with ATCC Vero 76 cells grown to 90–100% confluency were used for plaque assay. Dilutions were added to wells in duplicate, and neat samples were added in triplicate at 200 µL per well. Plates were incubated at 37 ± 2 °C and 5 ± 1% CO_2_ and rocked every 15 min. After one hour, cells were overlaid with 2 mL of 1.2% agarose mixed 1:1 with BME with 10% heat inactivated FBS and 2% pen/strep. Plaques were then incubated 24 ± 4 h at 37 ± 2 °C and 5 ± 1% CO_2_. A total of 2 mL of neutral red overlay (1.2% agarose mixed 1:1 with BME with 10% heat-inactivated FBS and 2% pen/strep and 4% of total volume neutral red vital stain (Quality Biological, Gaithersburg, MD, USA, Cat: 115-341-061) was added to wells and allowed to incubate for 18–24 h prior to visualization and quantitation.

### 2.6. Tissue Processing of Inactivated Samples for RNA Extraction

Inactivated samples were removed from formalin or glut/PFA. Each cube was then washed three times with PBS (Gibco, Miami, FL, USA, 10010-023). After three washes, the cube was minced using a scalpel. Minced tissue was weighed to verify that samples were less than 100 mg per manufacturer’s recommendations. Samples were then added to a sterile 2 mL RNase/DNase-free tube with 2.4 mm metal hard tissue grinding beads (VWR, St. Louis, MO, Cat: 10158-604). If tissues were more than 100 mg, samples were split between multiple tubes to obtain less than 100 mg per tube based on guidance from the Federal Select Agent Program [1]. A total of 900 µL of Qiazol (Qiagen, Germantown, MD, USA, Cat: 79306) and 2 µL of carrier RNA (Molecular Research Center MRC, Cincinnati, OH, Cat: PC-152) were then added to each tube. Tissue was homogenized using a bead homogenizer (VWR, St. Louis, MO, Cat: 35377) at setting 5 for 30 s. If samples were not uniformly homogenized, the homogenization step was repeated a second time. Samples were incubated at room temperature for five minutes followed by a centrifugation step to pellet the debris. Supernatants were transferred to a fresh tube and centrifuged a second time to remove any remaining debris. Supernatants were collected and then frozen at −80 °C ± 10 °C until RNA extraction was performed.

To prepare the positive controls, approximately 70 mg of naïve NHP brain tissue was spiked at the center with a known concentration of EEEV in 20 µL volume (1000 PFU, 100 PFU, 10 PFU, or 1 PFU). Samples were then added to a sterile 2 mL RNase/DNase-free tube with 2.4 mm metal hard tissue grinding beads (VWR, St. Louis, MO, Cat:10158-604). A total of 900 µL of Qiazol (Qiagen, Germantown, MD, USA, Cat:79306) and 2 µL of carrier RNA (Molecular Research Center MRC, Cincinnati, OH, Cat: PC-152) were then added to each tube. Tissue was homogenized using a bead homogenizer (VWR, St. Louis, MO, Cat: 35377) at setting 5 for 30 s. If samples were not uniformly homogenized, the homogenization step was repeated a second time. Samples were incubated at room temperature for five minutes followed by a centrifugation step to pellet the debris. Supernatants were transferred to a fresh tube and centrifuged a second time to remove any remaining debris. Supernatants were collected and then frozen at −80 °C ± 10 °C until RNA extraction was performed.

### 2.7. RNA Extraction of Inactivated Samples

Frozen (formalin or glut/PFA inactivated) homogenates were placed at 37 °C ± 1 °C until completely thawed. RNA was extracted from the samples prepared in Section 2.6 using an RNeasy Plus Universal Mini Kit (Qiagen, 73404). All steps were followed in accordance with the manufacturer’s instructions. If the entire tissue homogenate was larger than 100 mg, then the homogenate was split across multiple tubes for RNA extraction.

### 2.8. Safety Testing Passage of Inactivated RNA

Passage 1: ATCC BHK-21 cells were harvested from a confluent T175 flask, and cells were pelleted at room temperature for five minutes at 200× *g.* Supernatant was discarded, and the cell pellet was gently disrupted by flicking the tube. Cells were resuspended in 30 mL of Opti-mem (Gibco, 31985-062) and centrifuged at 200× *g* for five minutes at room temperature to wash. Again, the supernatant was removed, and the cell pellet was disrupted by gently flicking the tube. The pellet was resuspended in 0.5 mL of Opti-mem per cuvette (approximately 1.5 mL per T175 flask of confluent cells). Up to 200 µL of RNA from inactivated (formalin or glut/PFA) or control samples was added to a 4 cm gap sterile electroporation cuvette (BioRad, 1652088). If samples were greater than 200 µL in volume, additional electroporation samples were made until all RNA was added to cuvettes. BHK-21 cells (0.5 mL) were then added to each cuvette and inverted twice. Cells were then electroporated twice using a BioRad Gene Pulser II with capacitance Extender Plus (BioRad, Hercules, CA, USA, Cat: 1652660) at settings (220 V, 1000 µF capacitance) with a few seconds of rest in between pulses. Cells were gently transferred to a 15 mL conical tube containing 13 mL of complete media (MEM with 10% HI-FBS and 1% pen/strep) using a 1 mL pipet. Medium (500 µL) was then used to rinse the cuvette to collect remaining cells and placed into the conical. Samples were inverted twice to mix. If more than one cuvette was needed to electroporate pooled RNA samples, they were removed from cuvettes, pooled, and mixed prior to splitting into flasks. Cell-containing media were then transferred to a T75 flask and incubated at 37 ± 2 °C and 5 ± 1% CO_2_ (approximately 14 mL in total). CPE was observed for 72 h. After 72 h (or when CPE reached ≥ 80% for control samples), supernatants were collected and centrifuged at 500× *g* for five minutes at room temperature to remove cell debris. Supernatants were removed and added to new tubes and frozen at −80 °C until passage 2.

Passage 2: Supernatants were thawed at room temperature, and 7 mL (50% of sample) of the pass 1 sample and 7 mL of media was added to a new T75 flask of BHK-21 cells. CPE was observed daily for 72 h. After 72 h (or when control infected cells reached ≥ 80% CPE), supernatants were collected into a tube. Samples and controls were centrifuged at 500× *g* for five minutes at room temperature to remove cell debris. Supernatants were transferred to a new tube and frozen at −80 °C until ready for the plaque assay.

## 3. Results

### 3.1. Formalin and Glut/PFA Effectively Inactivate EEEV Particles in Tissue Samples

**Figure 3:**
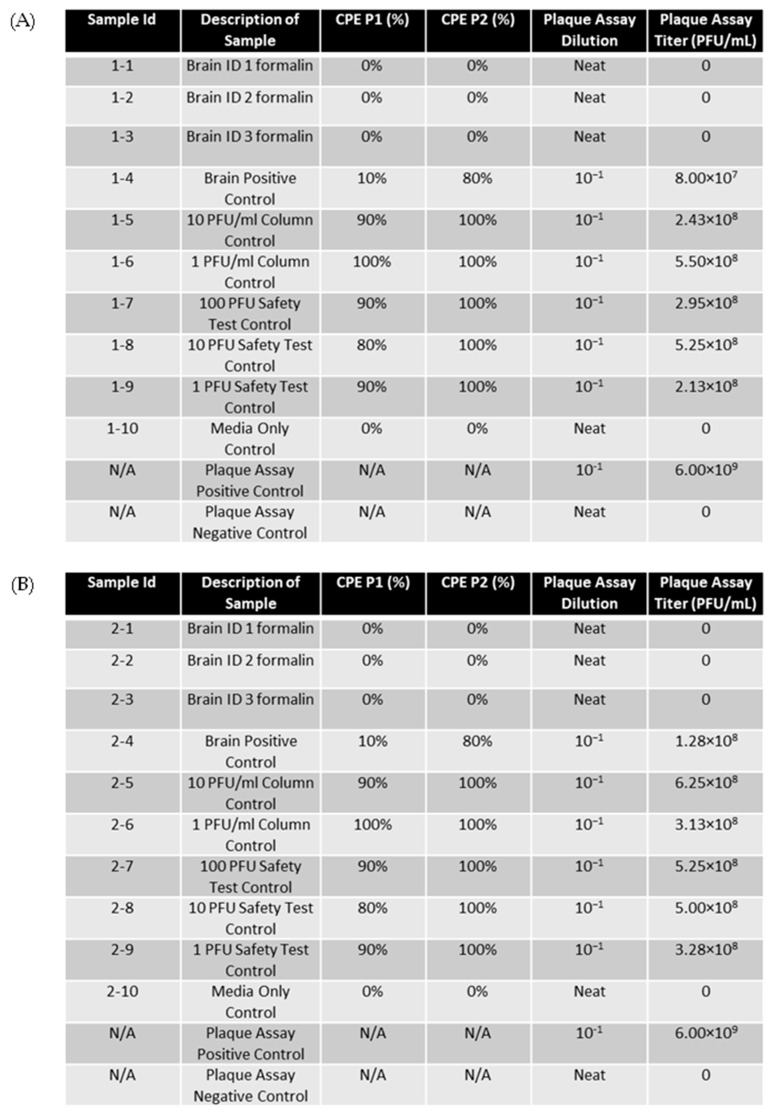

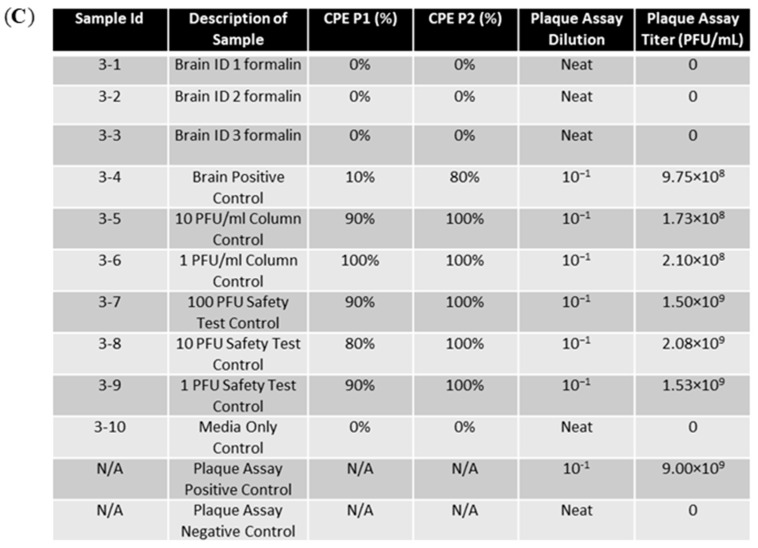
EEEV-infected NHP brains were fixed in 10% formalin and homogenized alongside positive control tissue samples. Centricon filtration was used to remove formalin, and supernatants were used to perform the first cell passage on ATCC Vero 76 cells for 72 h. A second passage was also performed for 72 h. CPE was observed and recorded daily. Column controls consisted of diluted virus run through a centricon filtration system, and the safety test control was performed by adding diluted virus directly to cell culture. Plaque assay was performed on formalin-fixed samples and control samples to determine EEEV titers. Stock virus was diluted and used as a plaque assay positive control. The limit of detection for the assay was 1 PFU/mL. (**A**) CPE and plaque assay data for replicate experiment 1. (**B**) CPE and plaque assay data for replicate experiment 2. (**C**) CPE and plaque assay data for replicate experiment 3.

Samples of EEEV-infected brain (10 m^3^) were fixed in 10% formalin for seven days and then homogenized alongside unfixed control tissue. Centricon filtration was used to remove residual formalin from the sample to prevent fixation/death of cells during safety testing. Supernatants were then passaged twice in ATCC Vero 76 cells, and cytopathic effects (CPEs) were observed daily for 72 h during both passages (Figure 3). For all three replicates, CPEs for formalin-fixed samples were less than 5%, with most of the samples showing a 0% CPE. Those that showed less than 5% were due to cell overgrowth and showed significantly less CPEs in comparison to positive controls. By the second passage all tissue controls, safety controls and column controls presented with a CPE of > 80% in all experiments; therefore, formalin fixation drastically decreased cellular cytopathic effects due to infection in comparison to positive controls as would be expected from an effective fixation method.

Plaque assays were performed on neat, fixed samples to detect any potentially infectious virus present in the samples (Figure 3). Virus stock was serially diluted from 10^1^ to 10^9^ and used as a positive control for assay performance. The results showed that no plaque-forming units developed in neat, fixed samples, whereas all controls presented with titers ≥ 10^7^ PFU/mL. These results demonstrate that the viral particles of EEEV in tissue are effectively rendered non-infectious upon fixation with formalin for seven days.

**Figure 4:**
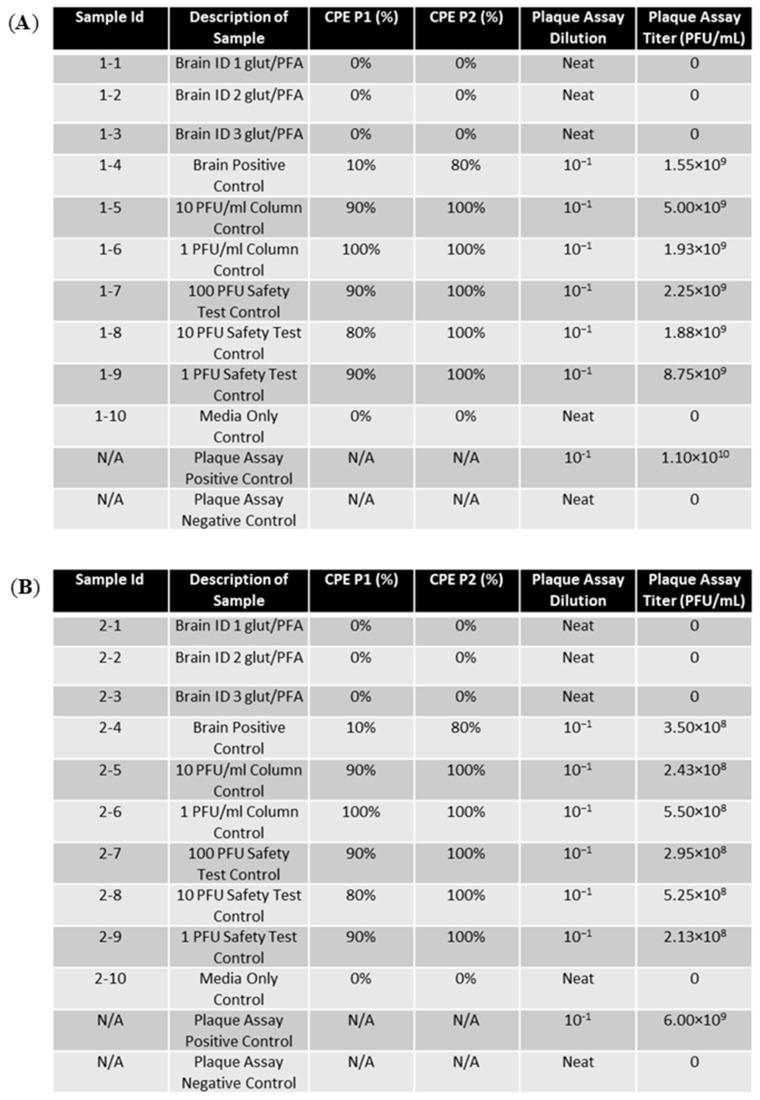

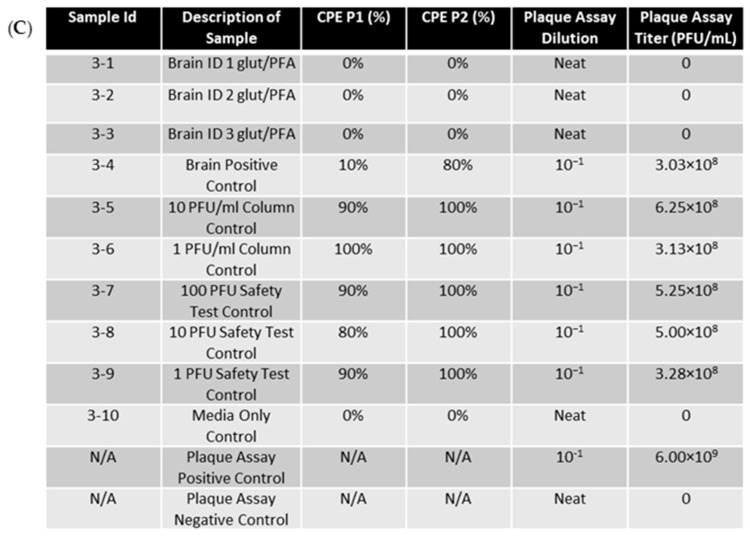
EEEV-infected NHP brains were fixed in glut/PFA and homogenized alongside positive control tissue samples. Centricon filtration was used to remove formalin, and supernatants were used to perform the first cell passage on ATCC Vero 76 cells for 72 h. A second passage was also performed for 72 h. CPE was observed and recorded daily. Column controls consisted of diluted virus run through a centricon filtration system, and the safety test control was performed by adding diluted virus directly to cell culture. Plaque assay was performed on glut/PFA fixed samples and control samples to determine EEEV titers. Stock virus was diluted and used as a plaque assay positive control. The limit of detection for the assay was 1 PFU/mL. (**A**) CPE and plaque assay data for replicate experiment 1. (**B**) CPE and plaque assay data for replicate experiment 2. (**C**) CPE and plaque assay data for replicate experiment 3.

Samples of EEEV-infected brain tissue NHP were trimmed to a 0.5 cm^3^ cube and placed in 2.5% glutaraldehyde/2% paraformaldehyde in 0.1 M cacodylate buffer (glut/PFA) for two days. The fixed tissue samples and unfixed positive control samples were homogenized and run through a centricon filtration system to remove glut/PFA. The samples were passaged twice on ATCC Vero 76 cells for 72 h per passage. The CPE was observed daily and recorded (Figure 4). Cells passaged with glut/PFA fixed cells presented with a 0% CPE for both passages and paralleled the media-only control. In contrast, unfixed positive tissue controls, column controls, and PFU controls produced an 80–100% CPE in Vero cells for both passages.

Neat supernatants from the second passage of glut/PFA fixed samples were then run on a plaque assay to measure titers (Figure 4). No plaques were produced for any fixed samples indicating that the virus in these supernatants was inactivated. The unfixed controls (tissue, PFU, and column control) all produced titers of greater than 10^8^, validating the proper inactivation of the fixed samples.

For both experiments, three different controls were used. The first was unfixed EEEV-infected tissue that acted as an experimental tissue control and was processed in parallel to the fixed tissue samples. This sample verified that the virus was in the initial tissue samples that were fixed. The second was the column controls that were created by diluting the virus to 1000 PFU, 100 PFU, 10 PFU, and 1 PFU and ran through the column. These positive controls verified that the virus was not being removed by the column during processing. Finally, a plaque assay control was created by serially diluting stock virus and inoculating plaque assay wells to verify the proper performance of the plaque assay procedure.

### 3.2. Formalin and Glut/PFA Also Inactivate Infectious RNA Genomes in EEEV-Infected Tissue

**Figure 5:**
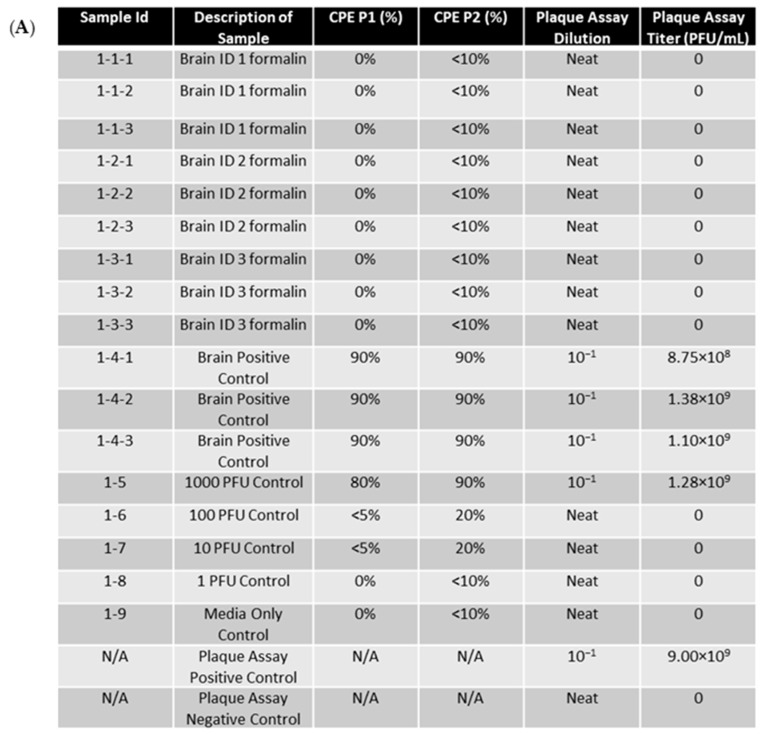

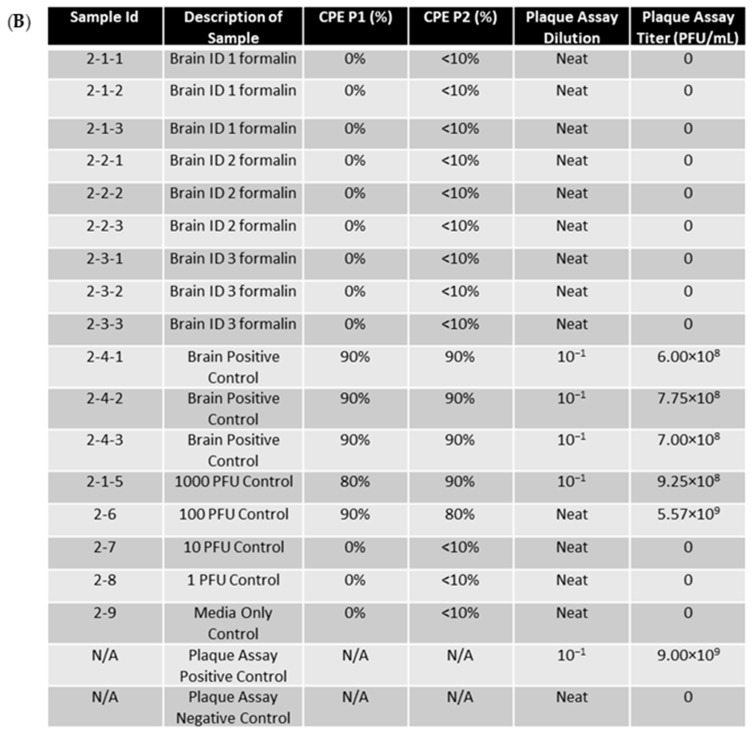

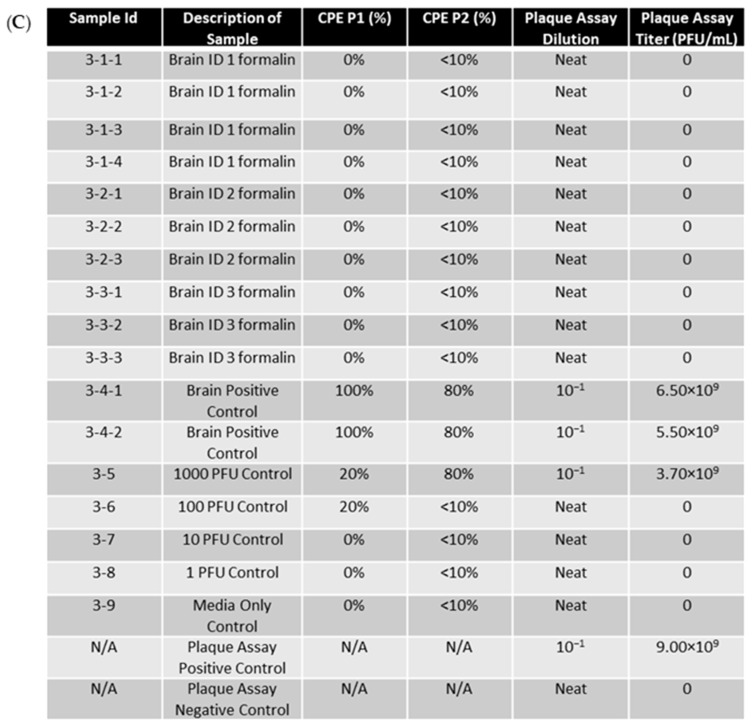
EEEV-infected NHP brains were fixed in 10% formalin and homogenized alongside positive control tissue samples. RNA extraction was then performed on samples. RNA was then electroporated into BHK-21 cells. RNA samples that were more than 0.5 (electroporation volume) were split into separate samples (ex: 3-1 into 3-1-1, 3-1-2, and 3-1-3) for electroporation into cells. Cells were then placed in flasks for 72 h as a first passage. A second passage was also performed for 72 h. CPE was observed and recorded daily. PFU controls were created by adding diluted stock virus at the designated PFU directly to cell culture. Plaque assay was performed on RNA extracted from formalin-fixed samples and control samples to determine if genomic replication and infectious virus production were occurring. Stock virus was diluted and used as a plaque assay positive control. The limit of detection for the assay was 1 PFU/mL. (**A**) CPE and plaque assay data for replicate experiment 1. (**B**) CPE and plaque assay data for replicate experiment 2. (**C**) CPE and plaque assay data for replicate experiment 3.

Brain tissue (1 cm^3^) from EEEV-infected NHPs was placed in 10% formalin for seven days. Unfixed control tissue and formalin fixed tissues were then homogenized, and formalin was removed utilizing centricon filtration. Supernatants were then collected and utilized to perform RNA extraction to isolate EEEV genomic RNA. Electroporation was then used to permeabilize BHK-21 cells, allowing for the introduction of the extracted genomic RNA into the cell. BHK-21 cells were utilized over other cell lines for viral RNA introduction via electroporation because they are easily transfected with viral RNA, allow for effective Alphavirus replication, and tolerate electroporation well [14,15]. Electroporated cells were incubated in a T-75 flask for 72 h, and the CPE was observed daily (Figure 5). Supernatants from passage 1 were then used for a second 72 h passage on BHK-21 cells. The CPE was again observed and recorded daily. The results showed that the CPE of cells infected with RNA extracted from formalin-fixed brains was comparable to that of the media control (0% for passage 1 and <10% for passage 2). In contrast, non-fixed tissue controls produced a CPE of 90–100% for passage 1 and 80–90% for passage 2. The CPE was also seen in all three 1000 PFU safety testing control samples, and an increased CPE was seen in some replicates of the 100 and 10 PFU safety control. The media-only safety control, which acted as a negative control, and the 1 PFU safety control produced no CPE or a CPE consistent with cell culture overgrowth. This suggests that RNA extracted from formalin-fixed tissue is not cytopathic, unlike RNA extracted from unfixed tissues.

A plaque assay was performed on the supernatants of the second passage to determine viral titers (Figure 5). Supernatants from cells electroporated with RNA isolated from fixed tissue presented with zero plaques in all replicates, demonstrating that formalin fixation effectively inactivates EEEV RNA extracted from fixed tissue samples. EEEV RNA extracted from unfixed positive control tissue produced titers >10^8^ PFU/mL. These data show that 10% formalin fixative can effectively be used for the inactivation of EEEV-infectious RNA. The 1000 PFU-positive control also produced countable titers using plaque assay techniques.

**Figure 6:**
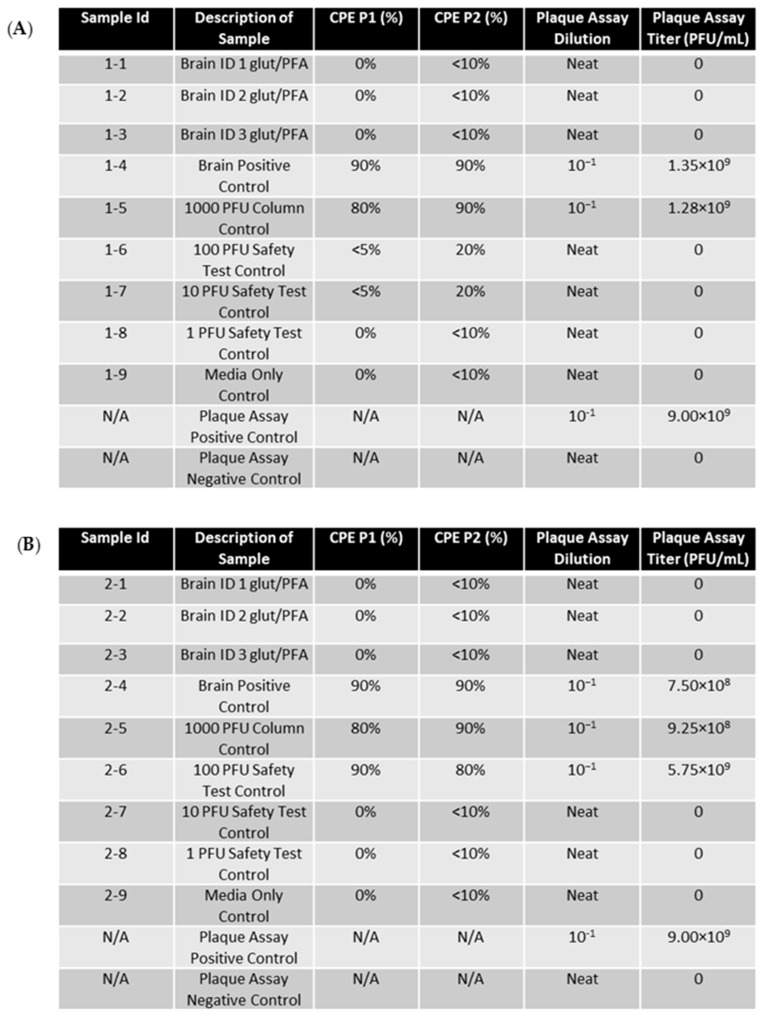

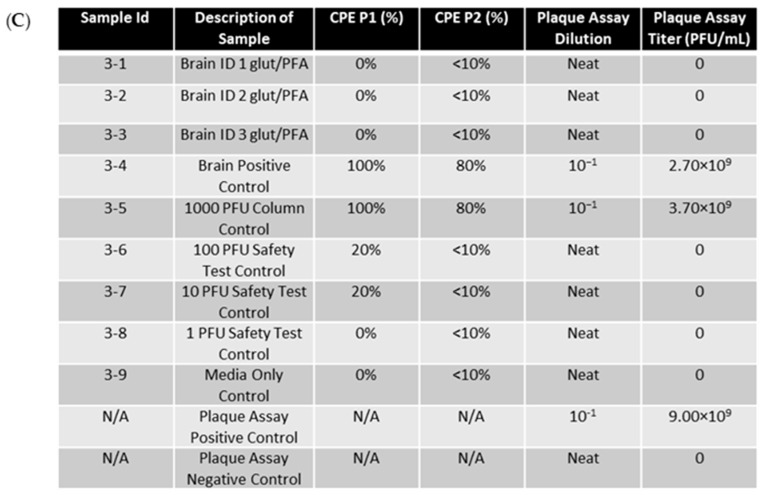
EEEV-infected NHP brains were fixed in glut/PFA and homogenized alongside positive control tissue samples. RNA extraction was then performed on samples. RNA was then electroporated into BHK-21 cells. Cells were then placed in flasks for 72 h as a first passage. A second passage was then performed for 72 h. CPE was observed and recorded daily. PFU controls were created by adding diluted stock virus at the designated PFU directly to cell culture. Plaque assay was performed on RNA extracted from formalin-fixed samples and control samples to determine if genomic replication and infectious virus production was occurring. Stock virus was diluted and used as a plaque assay positive control. The limit of detection for the assay was one PFU/mL. (**A**) CPE data for replicate experiment 1. (**B**) CPE and plaque assay data for replicate experiment 2. (**C**) CPE and plaque assay data for replicate experiment 3.

EEEV-infected NHP brain was trimmed to a 0.5 × 0.5 × 0.5 cm cube and placed in glut/PFA fixative for two days. The entire pieces of tissue samples and unfixed control tissue were homogenized, and supernatants were used for RNA extraction. All extracted RNA was electroporated into BHK-21 cells, allowing for bypass of entry and fusion steps. The BHK-21 cells were then allowed to grow as the first passage of the virus. Supernatants were then used to pass again in BHK-21 cells. Each passage was performed for 72 h, and the CPE was observed daily (Figure 6). The CPE of cells electroporated with glut/PFA fixed RNA were 0% and <10%, respectively, for passage 1 and passage 2. Since these values were reflective of the media-only control, the <10% seen in passage 2 was due to overgrowth of cells. The unfixed tissue controls and 1000 PFU control presented with CPEs of 80–100% for both passages. These results confirm that glut/PFA fixation prevents CPE caused by EEEV genomic RNA.

A plaque assay was used to quantify the titers of the neat, glut/PFA fixed RNA infected samples (Figure 6). No plaques were detected in any of the fixed RNA infected samples, showing the effective inactivation of the EEEV genomic RNA. The tissue positive control, 1000 PFU-positive control, and plaque assay-positive control all presented with titers >10^8^ PFU/mL. Plaques produced by the positive controls and not produced by the fixed samples validate that glut/PFA can effectively be utilized to inactivate EEEV RNA.

## 4. Discussion

The aforementioned techniques validate the ability to rapidly inactivate EEEV in tissue samples utilizing two forms of chemical inactivation: 10% formalin and glut/PFA. The studies demonstrated that EEEV particles and genomic RNA could be inactivated by glut/PFA after two days of fixation and after seven days in 10% formalin. The methods provide techniques to effectively remove fixative reagents from viral products within the supernatant that could kill cells during passaging. The methods also provide appropriate means to actively test the inactivation of replicative genomes of positive-sense RNA viruses utilizing electroporation to artificially bypass entry and fusion steps. These methods should be applicable for validating the inactivation of other positive-sense RNA viruses in accordance with CDC requirements. There is a possibility that differences in the genomes of positive-sense RNA virus families could render these techniques less fruitful outside of the Togaviridae family; therefore, it is essential to extensively validate other FSAP-regulated non-Togaviridae positive-sense RNA viruses to further establish the universality of these techniques.

Two other techniques have been validated to inactivate alphavirus RNA: viral RNA post-fragmentation and cDNA production with RNase A and H treatment. These inactivation methods were used in conjunction with transfection, allowing entry of the genomic RNA into the cell as suggested by the CDC [14]. The two techniques demonstrated in this paper expand the arsenal of inactivation options for positive-strand RNA viruses. Glut/PFA and 10% formalin are particularly beneficial for techniques that require the RNA, cell, or other molecular components to remain intact for pathological examination or electron microscopy [15,16]. These chemical inactivation procedures also provide rapid results, allowing samples to be rapidly removed from the suite for processing. In conclusion, this research provides essential methods to improve efficiency and biosafety when working with positive-strand RNA viruses in containment.

## Figures and Tables

**Table 1:** Positive-strand RNA viruses designated as select agents by the Federal Select Agent Program.

FSAP Regulated Positive-strand RNA
Classical swine fever virus
Eastern equine encephalitis virus (North American genotypes)
Foot-and-mouth disease virus (FMDV)
Kyasanur Forest disease virus
Omsk hemorrhagic fever virus
SARS-associated coronavirus (SARS-CoV)
Swine vesicular disease virus
Tick-borne encephalitis complex flavivirus (far Eastern subtype and Siberian subtype)
Venezuelan equine encephalitis virus subtypes (IAB and IC)

## Data Availability

The data presented in this study are provided within this article and available upon request from the corresponding author.
